# Recreational landscape perception and pro-environmental behavior: the mediating roles of place identity and pro-environmental behavioral spillover

**DOI:** 10.3389/fpsyg.2025.1616154

**Published:** 2025-09-11

**Authors:** Ke Zhang

**Affiliations:** School of Fine Arts and Design, Chuzhou University, Chuzhou, China

**Keywords:** recreational landscape perception, place identity, pro-environmental behavior, S-O-R theory, spillover effect, tourism

## Abstract

**Introduction:**

Tourism significantly contributes to global economies but also intensifies environmental pressures at destinations. Promoting tourists’ pro-environmental behavior (PEB) is therefore crucial for sustainable tourism. Recreational landscape perception (RLP), a multidimensional and integrative concept, may shape visitors’ emotional connections and subsequent behaviors. However, few studies have examined its combined effects on both place-specific and general PEB, or the underlying psychological pathways. This study applies the Stimulus-Organism-Response (S-O-R) model to investigate how RLP influences PEB through place identity and behavioral spillover.

**Methods:**

A visitor survey was conducted in Langya Mountain National Forest Park during peak season, yielding 457 valid responses. Recreational landscape perception, place identity, and both place-specific and general PEB were measured using established Likert scales. Structural equation modeling with bootstrapping was applied to test the proposed relationships and mediation effects.

**Results:**

The results showed that: (a) RLP significantly and positively influences place-specific PEB through place identity; (b) RLP significantly and positively influences general PEB through place identity; and (c) RLP significantly and positively influences general PEB through both place identity and place-specific PEB.

**Discussion:**

The findings demonstrate that RLP, as a multidimensional perception of destination landscapes, significantly enhances both place-specific and general PEB, with place identity and behavioral spillover acting as key mediators. Theoretically, this study advances the S-O-R framework by integrating natural, cultural, and experiential dimensions of landscape perception into a unified model, and by empirically validating the dual mediation pathway from destination-specific experiences to broader environmental actions. Practically, the results offer evidence-based guidance for sustainable tourism planning-highlighting that enhancing visitors’ holistic landscape experiences can strengthen emotional bonds, stimulate on-site conservation behaviors, and encourage the adoption of eco-friendly habits in daily life.

## 1 Introduction

The global tourism industry has expanded rapidly, with international tourist arrivals reaching 1.4 billion in 2024, and global tourism revenue totaling 1.6 trillion USD (UNWTO, 2025). Although a significant contribution to the economy, it is also accompanied by serious resource wastage problems and environmental degradation in tourist destinations (Kalayci and Hayaloğlu, 2019). For example, more than 200 million tourists travel to the Mediterranean Sea each summer, leading to a 40% rise in plastic waste in the ocean (United Nations Environment Program, 2019). Inappropriate tourist behaviors exhibited by tourists can adversely affect the natural environment of destinations, including vegetation destruction (Thanh and Asanka, 2025), illegal collection of flora and fauna specimens (Kapera and Kapera, 2021), disruption of wildlife habitats (Newsome, 2021), and pollution (Zeng et al., 2021).

In response, many countries have implemented punitive measures to combat environmentally harmful tourist behavior. For example, the U.S. government has introduced laws such as the Clean Water Act, Clean Air Act, and Endangered Species Act since 1940, imposing fines and criminal penalties for environmental offenses. Similarly, China has enacted legislation like the Tourism Law of the People’s Republic of China (2013) and the Interim Measures for the Administration of Records of Uncivilized Behavior of Tourists (2015). Although these approaches have helped to address tourism’s issues, their effectiveness is still controversial. Tourists’ anonymity may prevent them from observing environmental regulations in unfamiliar environments, causing deviant behavior (Geng et al., 2016; Oliver et al., 2021). To address these challenges, researchers have advocated shifting from reliance on regulations and punitive measures to promoting pro-environmental behavior (PEB) as a long-term solution (Salla et al., 2025). This approach focuses on the psychological and situational factors that encourage individuals to voluntarily take environmentally responsible actions. In the tourism context, PEB refers to voluntary actions that can reduce the negative impact of tourism activities on the environment, including reducing resource consumption, choosing environmentally friendly transportation, and participating in ecological protection activities (Wu et al., 2021). Empirical studies have shown that PEB plays a dual role: it not only mitigates direct environmental impacts but also generates spillover effects, fostering greater ecological awareness among the public (Halpenny, 2010). However, much remains unknown about how tourists’ environmental attitudes and behaviors are shaped by tourism environments, and how these behaviors may spill over into broader contexts beyond the immediate travel setting.

The factors that affect PEB can be analyzed in two main categories. The first includes the theory of reasoned action (Ajzen and Fishbein, 1980) and the theory of planned behavior (Ajzen, 1991). According to these ideas, people make decisions by weighing benefits against costs, and that their actions largely depend on their behavioral intentions. The second direction concentrates on social norms and includes the norm activation model (Schwartz, 1977) and value-belief-norm theory (Stern, 2000). However, these perspectives emphasize how socially responsible actions, such as PEBs, influence a person’s social duty and beliefs. These theories do not completely account for PEBs’ avoiding emotional or personal objectives, such as tourists who take the initiative to clean up garbage left by others or purchase more expensive but eco-friendly memorabilia. Explanations for such behaviors may lie in the influence of external environments. According to environmental psychology, an individual’s perception of the environment can substantially impact their emotions (Ruesch, 1965). Positive emotions, in turn, can enhance creative thinking and expand cognitive information, and then respond more appropriately to behaviors in a specific environment (Zelenski and Desrochers, 2021).

Recent research has begun to explore how positive emotions contribute to PEBs in tourism contexts (Li et al., 2023; Su et al., 2025), yet the origins of these emotions are still inadequately comprehended. In this context, this study applied the stimulus-organism-response (S-O-R) model (Mehrabian and Russell, 1974), which considers the external environment as a prerequisite for the generation of positive emotions and provides a theoretical framework that integrates the external environment (stimulus), emotional states (organism), and behavioral reactions (response). Although previous studies have identified the role of external environmental stimuli in initiating PEB, they often focus on isolated factors. For example, Lin and Lee (2020) found that immersive recreational activities, such as cultural exhibitions, enhance landscape perception and foster environmental responsibility, leading to place-specific PEB. Similarly, Baird et al. (2022) linked awe-inspiring natural vistas to heightened conservation actions. However, much of the existing research tend to treat natural and cultural components separately, overlooking their synergistic effects.

In summary, despite increasing interest in sustainable tourism, limited attention has been paid to how tourists’ perceptions of recreational landscapes translate into PEB across diverse contexts. Existing research often examines natural or cultural stimuli in isolation, neglects the integrated effects of multi-dimensional landscape perceptions, and few studies have explored how these perceptions influence tourists’ psychological pathways to environmental protection during and after their visit. Addressing these gaps, this study adopts Stimulus-Organism-Response (S-O-R) model to investigate how RLP affects both place-specific and general PEBs, emphasizing the mediating roles of place identity, on-site protective behaviors, and their spillover effects on daily life. RLP is conceptualized here as a holistic concept that includes tourists’ comprehensive perceptions of the natural elements, cultural characteristics, spatial quality, and service-related attributes that together shape their emotional connection to the destination and influence subsequent behavioral intentions. The findings can inform the landscape features that enhance visitors’ emotional connection to destinations, inspire on-site pro-environmental actions, and encourage the integration of pro-environmental habits into daily life. These insights provide evidence-based strategies for long-term sustainable tourism management and policy development. To achieve these aims, we conducted a quantitative survey in Langya Mountain National Forest Park, collecting data from visitors during the peak season, and applied structural equation modeling (SEM) with bootstrapping to empirically test the proposed model and hypotheses.

## 2 Literature review

### 2.1 Pro-environmental behavior

Research on pro-environmental behavior (PEB) emerged in the 1970s, deeply influenced by social and environmental psychology. PEB is broadly defined as the conscious pursuit of minimizing the negative impacts of one’s actions on the natural and built world (Kollmuss and Agyeman, 2002). A related but more specific concept is environmentally responsible behavior (ERB). ERB refers to explicit, observable actions individuals consciously take to address environmental problems or directly protect the natural environment, such as avoiding littering, reducing energy and water consumption, recycling, following ecological guidelines, and participating in conservation activities (Borden and Schettino, 1979; Han et al., 2018; Qiu et al., 2023). While PEB is a broader, outcome-oriented concept encompassing all actions (including intentions, attitudes, and habits) that produce environmental benefits relative to alternatives, ERB focuses on specific, context-specific behaviors that have a direct environmental impact. In many subsequent studies, these terms have been used interchangeably, but in this study, PEB is used as an umbrella concept that covers both place-specific and general environmentally beneficial actions.

Research on factors affecting PEB can be divided as follows: living environment (Netuveli and Watts, 2020), demographic variables (Collado et al., 2019), social norms (Reynolds et al., 2015), daily habits (Liu et al., 2020), positive emotions (Chatelain et al., 2018), etc. In the context of tourism, researchers have focused on tourists’ PEBs, which include all activities of tourists during tourism that aim to minimize the negative impacts on the destination’s ecology and the local community (Steg and Vlek, 2009). Scholars have found that people tend to show lower PEB tendencies in tourism contexts compared to their everyday environments (Carneiro et al., 2022). This scenario identifies two main influences on PEB: one is the external environment perception, such as animal contact (Clark et al., 2019), natural environment perception (Baird et al., 2022), digital media (Chen et al., 2024; Liao, 2024); and the second is internal motivations of individuals, such as place attachment (Halpenny, 2010), nostalgia (Wu et al., 2020), anticipatory emotions (Han et al., 2018), norms and morals (Wu et al., 2020), among others. It can be seen that previous studies have focused on the direct impact of particular natural factors or specific emotions on PEB. However, as previously stated, tourists’ perceptions of a tourism destination are often comprehensive. This comprehensive perception-termed “recreational landscape perception”-encompasses not only natural elements (e.g., scenic beauty) but also cultural and social interactions (e.g., historical sites, recreational activities), forming a holistic psychological experience that integrates multisensory stimuli and cognitive evaluations (Potschin and Haines-Young, 2006; Hansen, 2021). The mechanism by which this integrated RLP affects PEB has not been elaborated in existing research.

Moreover, researchers have explored the spillover effect of PEB, in which environmentally responsible behaviors demonstrated in one setting may transfer to other areas (Halpenny, 2010; Yoon et al., 2024). Based on this, this study proposes that RLP positively influences tourists’ PEB at the recreation space, which then carries over into their everyday environmental behaviors. Consequently, recreational landscapes can be strategically utilized to influence visitors’ perspectives, encourage their PEBs at the site, and drive similar behaviors in their daily lives through spillover effects, ultimately promoting environmental conservation behaviors on a broader scale.

### 2.2 Recreational landscape perception

RLP consists of three concepts: “landscape” refers to a dynamic human–environment system combining natural and human-shaped elements, perceived and valued through cultural and experiential lenses (Bender, 2024). “Perception” is the process by which people receive and interpret information (Phipps, 1988). Zube et al. (1982) structured landscape perception into three basic elements: people, landscape, and interactions. Then, Daniel (2001) defined landscape perception as the interaction between physical features of the landscape and human perceptual judgment. “Recreation” here refers to leisure activities that take place in and depend on the natural environment (Morse et al., 2022). Purely aesthetic natural landscapes may not sustain interest, while recreation enhances visitors’ interest and experience by increasing interaction and participation. Hansen (2021) reviewed the coupling of the concepts of “recreation” and “landscape,” stating that “Recreational landscape” is an important part of the landscape of nature tourism destinations, including both natural and cultural-social elements. Lin and Lee (2020) empirically quantified how recreation experiences affect visitors’ environmentally responsible behavior, highlighting the role of integrated natural, cultural, and spatial attributes in shaping perceptions. In summary, RLP represents a complex psychological process shaped by multidimensional interactions and influences, which covers the comprehensive experience of individual’s perceptions, understandings, and interactions with landscape elements in both natural and artificial environments. It involves not only the perception of physical attributes but also profound interactions within cultural, social, and emotional dimensions. Unlike “landscape appreciation” (Gordon, 2018), which often emphasizes aesthetic enjoyment, or “restorative environment perception” (Kaplan, 2001), which focuses on stress recovery and psychological restoration, RLP emphasizes an integrated, multidimensional perception that combines natural, cultural, spatial, and service-related attributes, and links these perceptions to place identity and behavioral outcomes.

Scientists have carefully studied the relationship between recreational activities and people’s environmental attitudes. According to some research, visitor’s PEBs increase when they engage in recreational experiences in natural environments (Lee and Jan, 2015). But Høyem (2020) claim that recreational activities alone do not promote PEB unless they are accompanied by emotional connections. This suggests that researchers should further explore how recreation experiences are internalized and transformed psychologically, and explore how these psychological processes influence tourists’ environmental responsibility and behavioral decisions. Understanding these mechanisms is crucial for developing mire effective environmental intervention strategies for tourist destinations.

### 2.3 Theoretical framework

O-R theory, developed by Mehrabian and Russell (1974), incorporates the concept of the organism into the traditional stimulus-response (SR) model, explaining how the external environment (stimulus) may provoke behavioral responses by influencing an individual’s internal state (organism). By focusing on the impact of an individual’s internal state in mediating between stimulus and response, the S-O-R theory provides a framework for understanding and predicting human behavior. An individual’s perception of a stimulus can trigger a sequence of cognitive and emotional changes that lead to specific behavioral outputs (Eroglu et al., 2003). This procedure may reveal how an individual’s behavioral decisions are affected by the external environment in a specific context through internal processing. The S-O-R theory is effective in explaining complex behavioral patterns in a variety of fields, including consumer behavior (Kim et al., 2020), environmental psychology (Tang et al., 2019), and tourism research (Su and Swanson, 2017).

Subsequent scholars have expanded the S-O-R framework to better capture the complex human-environment interactions. Bitner (1992) introduced the concept of “servicescape”, extending S-O-R beyond emotional responses to include cognitive and physiological reactions to physical surroundings. Jacoby (2002) further integrated cognitive and affective systems into the framework, emphasizing the role of long-term memory and prior experience in shaping organismal responses. Kim and Lennon (2013) expanded the model by incorporating both internal stimuli and external stimuli in online settings, demonstrating how cognitive and affective processes jointly mediate behavioral responses. These advances highlight the flexibility of the S-O-R model and its relevance in explaining environmental influences on behavior across diverse contexts, including the tourism.

In recent years, several researchers have applied and expanded the S-O-R model to examine factors influencing PEB in tourism contexts. Su and Xu et al. (2020) examined how a destination’s eco-friendly reputation influences ERB, extending the S-O-R framework to include tourists’ emotional responses and satisfaction as dual mediators between environmental stimuli and environmentally responsible actions. Qiu et al. (2023) further refined the model by incorporating cognitive imagery, affective imagery, and place attachment as organismal states linking destination source credibility to tourists’ ERB. Beyond tourism, Kim et al. (2020) applied the expanded S-O-R model in a virtual reality shopping context, demonstrating how technological immersion and realism act as additional stimuli to influence consumers’ cognitive and emotional states, thereby triggering behavioral responses. These studies highlight the adaptability of the S-O-R framework and its value in explaining complex environmental influences on behavior.

Building on these advancements, this study considers RLP as an external environmental input with a wide range of environmental characteristics. It reflects tourists’ holistic evaluation of their surroundings and can therefore be considered a “stimulus” within the S-O-R framework. Place identity, defined as an individual’s self-concept tied to a specific place through emotional attachment and psychological dependence (Proshansky, 1978), is positioned as the organism. This positioning is consistent with its role as a mediating psychological state that translates environmental perceptions into behavioral intentions.

In this study, for research purposes, PEB is classified into two distinct dimensions: place-specific PEB and general PEB. Place-specific PEB is defined as behaviors directly related to the destination, such as adhering to conservation guidelines or avoiding ecological damage. These behaviors are environmentally relevant and stem from direct interactions with the recreational landscape. In contrast, general PEBs refer to eco-conscious practices in daily life, such as energy conservation and waste reduction that occur outside of tourist sites and reflect internalized environmental values.

**Figure 1:**
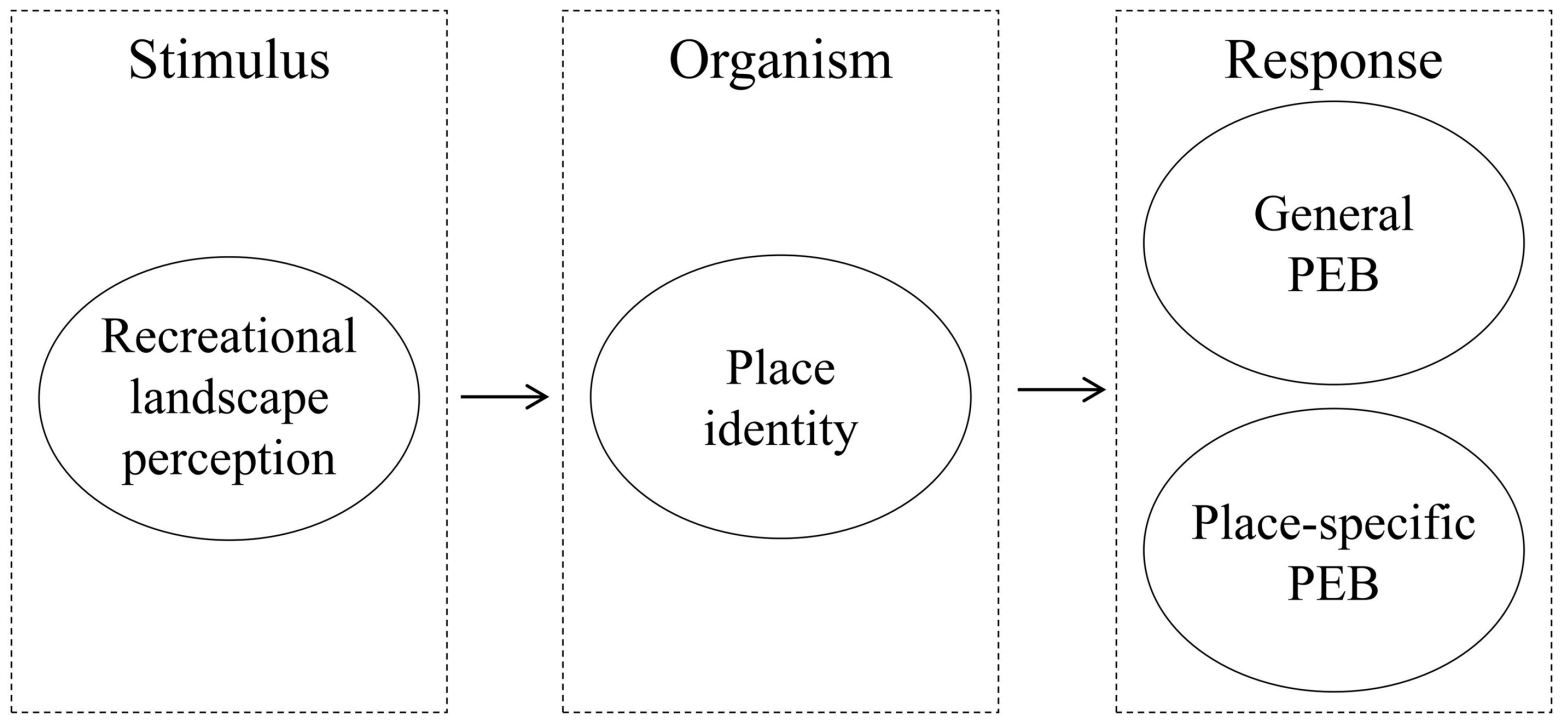
Theoretical model.

This framework systematically explores how tourists experience emotional and cognitive changes through the perception of landscape features, which ultimately result in specific environmental behaviors (Figure 1). By clarifying this causal process, the study not only provides a new perspective on understanding tourist behavior at destinations but also offers a theoretical foundation and empirical evidence to support the improvement of scenic area management and environmental protection strategies.

## 3 Hypotheses

### 3.1 Relationship between RLP and PEB

The formation of environmental attitudes is a long-term and complex process, shaped by a variety of experiences (Newhouse, 1990). Adults can develop or strengthen positive environmental views through their interactions with nature (Loeffler, 2004). However, increased environmental awareness is not solely the result of exposure to nature; more importantly, it is driven by specific recreation experiences (Lawrence, 2012) and active participation in recreational activities (Dunlap and Hefferman, 1975; Teisl and O’Brien, 2003). These activities provide visitors with opportunities to interact with nature, allowing them to directly experience environmental issues such as pollution and ecological degradation. Such direct participation helps to enhance environmental awareness (Lin and Lee, 2020). Recreational landscapes are not only part of the environment but also a powerful trigger for environmental awareness. In this process, RLP plays a vital role, promoting behavioral change through integrated, multi-dimensional experiences (Hansen, 2021). These experiences include contact with nature (Baird et al., 2022), cultural participation (Lin and Lee, 2020), and social cohesion (Đorđević et al., 2024).

This research may also verify whether the effect of RLP on park-specific PEB extends beyond the park, i.e., whether these behaviors have a positive spillover effect on broader environmental actions. Numerous studies have supported this phenomenon. For example, Halpenny (2010) demonstrated that attachment to a park increases a visitor’s PEB both within and beyond the park. Such attachment can transform into a broader concern for the environment, motivating individuals to engage in more eco-friendly behaviors. Similarly, Xu et al. (2020) found that tourism PEB positively affects domestic PEB, mediated by environmental identity. This finding confirms the research of Lanzini and Thøgersen (2014), which supports the positive spillover effect of PEB in different environments, indicating that improving location-specific PEB can effectively promote people’s environmental protection behaviors in the wider area.

Such spillover may occur through several interconnected psychological processes. First, identity activation happens when environmentally responsible actions at a destination deepen individuals’ sense of belonging and self-identification with nature. Second, value reinforcement takes place as immersive landscape experiences strengthen pro-environmental values, making them more likely to guide everyday decisions. Third, emotional resonance arises from meaningful and enjoyable interactions with the landscape, creating positive feelings that people seek to replicate in other contexts. Building on these insights, this study will explore whether RLP shapes park-specific PEB in ways that naturally extend to broader, sustained environmental actions beyond the tourism setting.

*H1*: RLP has a positive and significant effect on place-specific PEB.

*H2*: RLP has a positive and significant effect on general PEB.

*H3*: Place-specific PEB has a positive and significant effect on general PEB.

### 3.2 Relationship between place identity and RLP and PEB

Place identity refers to an individual’s self-perception in relation to the physical environment, arising from their use of and dependence on a place, together with a deep emotional and psychological connection to it (Proshansky, 1978). Place identity is the process by which people see themselves as belonging to a particular place through interactions (Hernández et al., 2010). It is a psychological bond that strengthens over time (Williams and Patterson, 1999). Many psychological theories about the relationship between people and their environment are based on this assumption (Peng et al., 2020). Thus, place identity extends beyond the functional use of a place, reflecting a deeper emotional attachment that fosters a sense of responsibility toward it (Relph, 2022). People with a positive emotional connection to a location are likelier to encounter PEB (Gosling and Williams, 2010).

Landscape perception also plays a key role in the formation of an individual’s place identity. Physiologically, people can be described as those who responder to environmental stimuli (Zube et al., 1982). Emotional psychology assumes that neural patterns of emotion have characteristic shapes in the brain that can be “recognized” in relevant shapes on the landscape, resulting in similar emotional responses (Clynes, 1969). This phenomenon suggests that people’s perception of the landscape involve more in-depth emotional and cognitive processing than just visual reception. The stimuli of the landscape create a strong bond between individuals and specific places. This bond can transform from a momentary sensory pleasure into a lasting “topophilia” (Tuan, 1990) and improve an individual’s feeling of place identity through emotional and cognitive interactions (Sobhaninia et al., 2023). In other words, enhancing the emotional connection between people and particular locations does not just involve extensive emotional and cognitive processing. Also, this emotional connection may lead to a greater understanding of the area.

In the tourism context, RLP fosters place identity through emotional immersion and cognitive meaning-making. Emotional engagement with natural, cultural, and social attributes fosters attachment and a sense of belonging, while cognitive interpretation aligns these perceptions with personal values. This goes beyond direct cognition or simple evaluation, as experiences are internalized into self-concept, forming the “organism” link in the S-O-R framework.

Furthermore, this emotional connection may extend to a broader recognition of the environment. Vaske and Kobrin (2001) proposed that as place identity increased, an individual’s attachment to the natural environment may expand to identification with the wider environment. Halpenny (2010) found that individuals may convert their appreciation of a specific place into a more abstract environment conceptions, thereby increasing the probability of participating in environmental conservation behaviors. Therefore, place identity and PEB in specific locations act as a bridge between landscape perception and PEB in other areas. In addition, recreational landscapes, as complex interactions of natural and cultural elements, provide immersive natural experiences that can not only cultivate emotional connections between visitors and the landscape, but also enhance awareness of environmental issues, motivating visitors to engage in PEB (Lin and Lee, 2020; Hansen, 2021). Compared with mere nature contact and recreational activities, landscape perception can more effectively promote visitors’ PEB through deeper emotional and cognitive mechanisms.

In summary, tourists’ perceptions of recreational landscapes enhance their identification with a particular place, and high levels of place identity encourage individuals to take action to protect and preserve the place (Moore and Graefe, 1994; Hernández et al., 2010), which directly affects PEB (Vaske and Kobrin, 2001; Halpenny, 2010) and may promote PEB in their daily environment. In this process, place identity and specific place PEB play important mediating roles.

*H4*: RLP has a positive and significant effect on place identity.

*H5*: Place identity has a positive and significant effect on place-specific PEB.

*H6*: Place identity has a positive and significant effect on general PEB.

*H7*: RLP positively and significantly influences general PEB through place identity.

*H8*: RLP positively and significantly influences place-specific PEB through place identity.

*H9*: RLP positively and significantly influences general PEB through place-specific PEB.

*H10*: RLP positively and significantly influences general PEB through place identity and place-specific PEB.

## 4 Methods

### 4.1 Study area and data collection

Langya Mountain National Forest Park is located in Chuzhou City, Anhui Province, China, covering a total area of 4,866.67 hectares with a variety of landscape types. The park has over 85% forest coverage, with a peak elevation of 312 meters above sea level, and is one of the first forest oxygen bar units in China, with rich natural resources. Langya Mountain Park is in the list of national forest parks, national key scenic spots, and national of 4A-level tourist area of China. The park is rich in cultural resources and diverse recreational spaces, including cultural relics and monuments, high-altitude adventures, cultural exhibition halls, and religious building areas, offering rich recreational experiences for visitors. According to statistics, the Park received more than 13,000 tourists with an income of 83,000 CNY during the 2024 Dragon Boat Festival holiday (Chuzhou Municipal Government, 2024).

The on-site questionnaire survey was conducted from March to May 2024, covering both weekdays and weekends, aiming to collect a representative sample of visitors during different periods. During the survey, we applied a systematic random sampling strategy along the park’s main hiking trails, inviting every tenth visitor to participate. The interval was determined based on estimated visitor flow and the capacity of the survey team (consisting of five trained enumerators) to ensure adequate spatial-temporal dispersion, minimize sampling bias, and maintain logistical feasibility in high-traffic conditions. Tourists were encouraged to scan a QR code (via Wenjuanxing APP, wjx.cn) to complete the questionnaire on their own phones, and a gift was offered upon completion. During this peak tourist season, the visitors to Mt. Langya are diverse in age, occupations, and income levels, resulting in a wide-ranging population. As of May 10, a total of 457 questionnaires has been completed. To determine an appropriate sample size, this study followed the guidelines proposed by Hair et al. (2010), which recommends that the minimum sample size in SEM should be at least 10 times the number of observed items associated with the most complex construct. The final questionnaire contained 41 observed items across all latent constructs. Therefore, the target sample size was set at a minimum of 410 valid responses. A total of 457 valid responses were collected, exceeding this threshold and ensuring sufficient statistical power for model estimation. Considering that some tourists might have limited time or might not answer the questionnaires carefully due to distractions, this study firstly excluded questionnaires with identical scores on the scale questions and then excluded those completed too rapidly (in less than 60 s) or too slowly (in more than 600 s). This screening can maximize data accuracy and questionnaire quality. However, it also led to the exclusion of numerous questionnaires, resulting in a relatively low validity rate of 86.2%, finally producing 392 valid questionnaires.

### 4.2 Measurements

Quantitative surveys are effective methods to measure the opinions of large numbers of people (Babbie, 1990), and in this research, data were collected by a self-administered questionnaire. The questionnaire was divided into four parts. The first part was the demographic profile of the tourists, including gender, age, education, annual income, and place of residence. The second part consisted of four items measuring the perception of the recreational landscape, designed by four scholars specialized in landscape studies and then tailored to Mt. Langya’s context. The items include tourists’ perceptions of natural and cultural landscapes, focusing on their emotional and cognitive responses to landscape elements. The third part consisted of three items measuring place identity, drawn from scales by Williams and Vaske (2003). The fourth part contained the items measuring PEBs, including three items for place-specific PEBs and three items for general PEBs, which were adapted from Halpenny’s (2010) scale. Considering the natural and cultural context of the site, the researchers adjusted the initial items accordingly. All scales used a 5-point Likert format, with scores ranging from 1 (strongly disagree) to 5 (strongly agree).

### 4.3 Data analysis

This study used SEM in AMOS 28.0 to evaluate the measurement and structural models. SEM was selected due to its suitability for testing complex theoretical frameworks involving latent variables and mediating pathways. Following Anderson and Gerbing’s (1988) two-step approach, we first assessed the reliability and validity of the measurement model through confirmatory factor analysis and then tested the structural model to assess the hypothesized relationships among constructs. All constructs in the model were treated as reflective indicators. Reliability was assessed through Cronbach’s alpha and composite reliability (CR), while convergent validity was evaluated using average variance extracted (AVE). Discriminant validity was assessed using the Fornell-Larcker criterion, which compares the square root of the AVE for each construct with the correlations between constructs. Discriminant validity is established when a construct’s AVE square root exceeds its inter-construct correlations. Model fit was based on multiple indicators, including the CFI, TLI, RMSEA, and SRMR. In addition, we used a bootstrapping method with 5,000 resamples to assess the significance of direct and indirect effects, providing robust support for the mediation hypothesis. The software used for analysis was IBM SPSS Statistics 28.0 and AMOS 28.0.

## 5 Results

### 5.1 Demographic profile of the respondents

The results of descriptive statistical analysis of 392 valid tourist samples according to control variables are presented in Table 1. The sample shows an equitable distribution of males and females. From the perspective of age composition, the sample covered a wide range of age groups, with a larger proportion of tourists aged 36 to 45 and 46 to 60. Tourists in these two age groups may have greater stability and financial ability in their families and occupations, thereby increasing their probability of participating in recreational activities. From the perspective of educational composition, college and undergraduate degrees constituted the majority (67.6%), which may indicate that well-educated tourists are more likely to participate in scenic areas rich in cultural and natural resources, or may be influenced by the presence of a university near Mt. Langya. The coverage from the perspective of income composition is relatively broad and well-distributed. It suggests that Mt. Langya, as a free and accessible nature park, is attractive to people of different levels of income. From the perspective of tourists’ residence, local visitors account for a majority (64.3%). This is related to the fact that Mt. Langya is located within the urban area of Chuzhou City, making it more accessible for local residents to visit frequently. A Mann–Whitney U test was conducted in SPSS, and it was found that there was no significant difference in PEB levels between local and non-local tourists (*U* = 15,998, *p* = 0.123), and the effect value was negligible (*r* = 0.078). In summary, the sample’s gender, age, education, income, and residence are widely distributed and balanced, thus comprehensively reflecting the attitudes and behaviors of tourists from diverse backgrounds. It provides a reliable dataset for later study and analysis.

**Table 1:** Demographic profile of the respondents.

Demographic Trait		Frequency (*n* = 395)	Percentage (%)
Gender	Male	193	49.2
	Female	199	50.8
Age	<18	5	1.3
	18–25	84	21.4
	26–35	69	17.6
	36–45	100	25.5
	46–60	96	24.5
	>60	38	9.7
Educational Level	High school or below	61	15.6
	College or University	265	67.6
	Graduate School	66	16.8
Annual Income	<40,000 CNY	116	29.6
	40,000 CNY-100,000 CNY	111	28.3
	>100,000 CNY and <200,000 CNY	123	31.4
	≥200,000 CNY	42	10.7
Current Address	Chuzhou City	252	64.3
	Non-Chuzhou City	140	35.7

### 5.2 Common method variance

Given that self-reported survey data may be susceptible to common method bias, it is important to assess whether Common Method Variance (CMV) significantly affects the relationships among constructs. CMV can inflate correlations and compromise the validity of causal inferences. In this study, several procedural precautions—such as item refinement, expert review, and anonymous participation—were implemented to mitigate CMV during the design phase. However, due to the uniformity of the measurement environment, CMV may still exist. To address this, the study used Harman’s Single-Factor Test to analyze the issue of CMV. First, an exploratory factor analysis was conducted on all the items, and the cumulative variance of all items was 75.872%, with the first factor accounting for 48.912%, which is below the 50% threshold (Podsakoff et al., 2003). This suggests that there is no significant CMV in the theoretical model.

### 5.3 Measurement model

Before analyzing the structural model, it is essential to assess the reliability and validity of the measurement model (Anderson and Gerbing, 1988). Factor analysis was conducted on the questionnaire using SPSS 27.0. The KMO value was 0.897, and the approximate chi-square value from Bartlett’s test of sphericity was 3121.840 with a *p*-value of 0.000, indicating that the sample data met the significance level. The measurement model was estimated using SPSS 27.0 and AMOS 28.0, calculating *p*-values, standardized estimates, squared multiple correlations (SMC), Cronbach’s alpha (*α*), Composite Reliability (CR), and Average Variance Extracted (AVE), with the findings presented in Table 2. The standardized factor loadings ranged from 0.581 to 0.930, Cronbach’s alpha ranged from 0.773 to 0.905, and all CR values exceeded 0.7, indicating the questionnaire’s acceptable reliability. Convergent validity was assessed using the AVE, and all AVE values exceeded 0.5, indicating good convergent validity of the measurement variables (Fornell and Larcker, 1981).

**Table 2:** Test results of the measurement model.

Construct	Observe variant	Items	Std. FL	SMC	α	CR	AVE
RLP	RLP1	The natural scenery of Langya Mountain is really stunning.	0.771	0.594	0.831	0.834	0.557
	RLP2	The pavilions at Langya Mountain provide me with an enjoyable experience.	0.744	0.554			
	RLP3	While visiting Langya Mountain, I can feel the unique local culture everywhere.	0.782	0.611			
	RLP4	During my visit, I came across some religious areas, and they really fascinated me.	0.685	0.469			
General PEB	GPEB1	In my daily life, I care about environmental issues and hope to contribute to environmental protection.	0.878	0.724	0.773	0.797	0.573
	GPEB2	In my daily life, I try to minimize my energy and water consumption.	0.781	0.619			
	GPEB3	In my daily life, I will try to buy organic food, even if it costs more, for the environment.	0.581	0.368			
Place-specific PEB	SPEB1	While visiting Langya Mountain, I will follow the environmental protection guidelines and avoid entering restricted areas.	0.879	0.803	0.889	0.896	0.743
	SPEB2	I will not damage or take away the flora and fauna on Mt. Langya when I visit the mountain.	0.930	0.822			
	SPEB3	During my visit to Langya Mountain, I will not harm or take any plants or animals from the mountain.	0.770	0.604			
Place identity	PI1	Langya Mountain is very special to me.	0.843	0.710	0.905	0.906	0.764
	PI2	Langya Mountain means a lot to me.	0.930	0.865			
	PI3	I love Langya Mountain.	0.846	0.716			

Std. FL = standardized factor loadings; α = Cronbach’s alpha; AVE = average variance extracted; CR = composite reliability.

Discriminant validity was measured by comparing the square root of the AVE with the correlation coefficient between the latent variables. Table 3 lists the square root of AVE of each latent variable, and the bold values represent these roots. The results show that the square root of the AVE of each latent variable is greater than the correlation coefficient with other variables, indicating that the measurement model has favorable discriminant validity. Reliability and validity tests indicate that the model has acceptable reliability, good convergent validity, and favorable discriminant validity.

**Table 3:** Correlation coefficients and AVE square root values of latent variables.

Construct	RLP	Place identity	Place-specific PEB	General PEB
RLP	**0.746**			
Place identity	0.683	**0.874**		
Place-specific PEB	0.449	0.463	**0.862**	
General PEB	0.692	0.715	0.635	**0.757**

The bold diagonal elements are the square roots of each AVE value; variable correlations are shown off-diagonal.

These results not only confirm the psychometric soundness of the measurement instruments but also support the theoretical distinctions among key constructs. Establishing this measurement quality is critical, as it lays a solid foundation for interpreting the causal paths in the structural model with confidence.

### 5.4 Structural model

**Figure 2:**
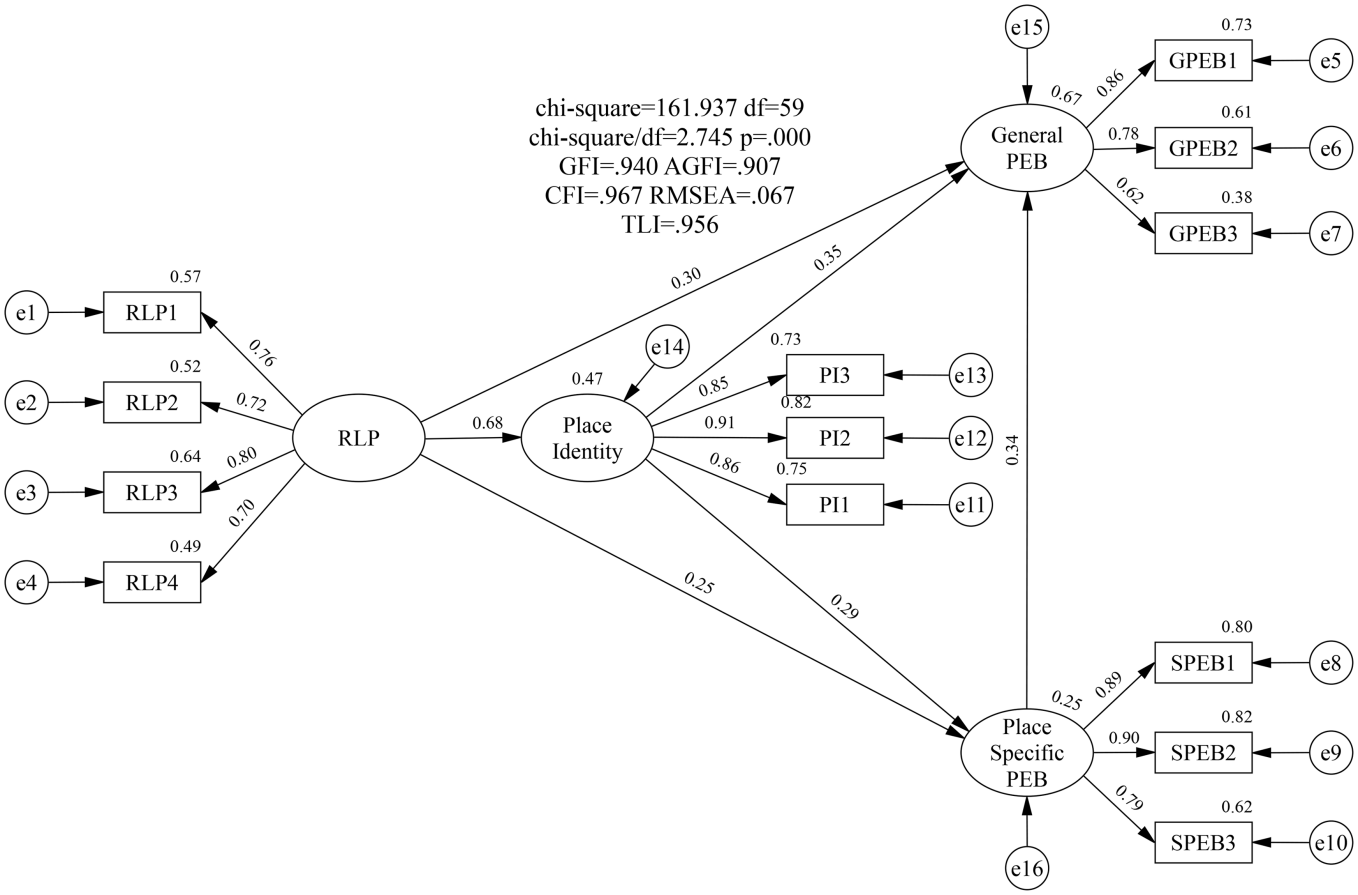
Structural model.

This study evaluated the structural model using SEM. Table 4 presents several common fit indices for the structural model, all within acceptable ranges. This indicates the model achieved good fit (Hair et al., 2010) and was appropriate for further analysis. The predictive accuracy of the structural framework was assessed by examining the R^2^ values: 46% of the variance in place identity, 66% in general PEB, and 24% in place-specific PEB were explained by the model (Hair et al., 2010). Figure 2 shows the standardized path coefficients. Table 5 shows the hypothesis testing of the structural model. The findings indicate that all six hypotheses of this study are supported: RLP positively and significantly affects both place-specific and general PEB; place-specific PEB positively and significantly affects general PEB; RLP positively and significantly affects place identity; and place identity positively and significantly affects both place-specific and general PEB.

**Table 4:** Goodness-of-fit test for the structural model.

Indices	Reference value	Model fit	Result
CMIN/DF	<3	2.745	Acceptable
GFI	>0.9	0.940	Acceptable
AGFI	>0.9	0.907	Acceptable
RMSEA	<0.08	0.067	Acceptable
SRMR	<0.08	0.045	Acceptable
CFI	>0.9	0.967	Acceptable
TLI (NNFI)	>0.9	0.956	Acceptable

**Table 5:** Results of hypothesis validation.

Hypothesis	Path	Direct Effect	*p*-value	Verification results
H1	RLP → place-specific PEB	0.248	0.002	Supported
H2	RLP → General PEB	0.299	***	Supported
H3	place-specific PEB → General PEB	0.337	***	Supported
H4	RLP → place identity	0.683	***	Supported
H5	Place identity → place-specific PEB	0.293	***	Supported
H6	Place identity → General PEB	0.355	***	Supported

***p < 0.001.

Results from the structural model provide strong empirical support for the proposed S-O-R framework to explain tourists’ PEB. The significant path from RLP to place identity suggests that tourists’ perceptions of the recreational landscape serve not only as external environmental stimuli but also as psychological cues that foster emotional and cognitive connections with the destination. This internalization process, reflected in place identity, in turn drives both place-specific and general PEB, confirming its mediating role. Furthermore, the significant path from place-specific to general PEB suggests behavioral spillover, whereby PEBs initiated by tourists during their visit extend into their daily lives. These findings validate the theoretical proposition that landscape perception transcends momentary aesthetic experiences and acts as a catalyst for behavior, and highlight the importance of emotional and identity-based mechanisms in promoting sustainable engagement beyond the immediate tourism context.

### 5.5 Mediating effects

This study used bootstrapping to test the significance of the mediating effect at a 95% confidence interval with 5,000 iterations (MacKinnon et al., 2004). Table 6 shows the results of the specific indirect effects. The indirect effect value for the path of RLP → place identity → general PEB was 0.235. The 95% confidence intervals for Bias-Corrected and Percentile were [0.114, 0.380] and [0.111, 0.380], respectively, both of which do not include zero, indicating that place identity plays a significant mediation role between RLP and general PEB. The indirect effect value for the path of RLP → Place Identity → place-specific PEB was 0.175, with 95% confidence intervals of [0.067, 0.324] and [0.060, 0.315] for Bias-Corrected and Percentile methods, respectively, both of which do not include zero, suggesting that place identity plays a significant mediation role between RLP and place-specific PEB. The indirect effect value for the path of RLP → place-specific PEB → general PEB was 0.081, with 95% confidence intervals of [0.021, 0.168] and [0.018, 0.160] for Bias-Corrected and Percentile methods, respectively, both of which do not include zero, suggesting that place-specific PEB plays a significant mediation role between RLP and general PEB. The indirect effect value for the serial two-mediator path of RLP → place identity → place-specific PEB → general PEB was 0.065. The 95% confidence intervals for Bias-Corrected and Percentile were [0.026, 0.127] and [0.022, 0.120], respectively, both of which do not include zero. This indicates that place identity and place-specific PEB play a significant chain mediation role between RLP and general PEB.

**Table 6:** Effect statement of mediating variable place attachment.

Hypothesis	Path	Indirect Effect (Mediation)	Product of Two Coefficients		Bootstrapping			
			SE	Z	Bias-Corrected 95% CI		Percentile 95% CI	
					Lower	Upper	Lower	Upper
H7	RLP → Place Identity→ General PEB	0.235	0.068	3.456	0.114	0.380	0.111	0.380
H8	RLP → Place Identity→ Place-specific PEB	0.175	0.066	2.652	0.067	0.324	0.060	0.315
H9	RLP → place-specific PEB → general PEB	0.081	0.036	2.250	0.021	0.168	0.018	0.160
H10	RLP → Place Identity→ place-specific PEB → general PEB	0.065	0.025	2.600	0.026	0.127	0.022	0.120

The mediating effect of place identity confirms that tourists’ emotional and psychological attachment to a destination plays a key role in shaping their PEB. This attachment reflects a deeper internalization process: positive landscape perceptions foster a sense of belonging and identification with the destination, thereby enhancing individuals’ intrinsic motivation to take pro-environmental actions. Specifically, this identity-based mechanism not only promotes PEB at the destination (place-specific PEB) but also extends to general PEB in daily life, suggesting that identity serves as a bridge between perceptions and broader behavioral patterns. Furthermore, the mediating effect of place-specific PEB suggests that immediate on-site behavioral engagement can reinforce and legitimize subsequent pro-environmental actions in everyday contexts. The presence of a significant sequential mediation path (RLP → place identity → place-specific PEB → general PEB) highlights the cumulative and cascading effects of emotional, cognitive, and behavioral responses. These findings provide strong support for the theoretical model and provide empirical evidence for the psychological mechanisms by which destination experiences shape long-term environmental commitment.

## 6 Discussion, conclusions and implications

### 6.1 Discussion and conclusions

The study used SEM to analyze empirical data, revealing the positive impact of RLP on tourists’ PEB and the partial mediating role of place identity in this process. The findings provide an empirical foundation for the design and management of recreational landscapes.

The results confirm that RLP has a significant positive impact on both place-specific PEB and general PEB. Tourists who experience high-quality recreational landscapes are more likely to adopt environmentally responsible behaviors at their destination (H1) and extend these sustainable practices into their daily lives (H2), indicating that positive environmental stimuli can directly promote both short-term and long-term environmentally responsible behaviors. RLP, a multifaceted concept that encompasses natural, cultural, and social dimensions, goes beyond traditional landscape perceptions which often focus primarily on visual aesthetics (Hansen, 2021). Early studies emphasized that contact with nature is a driving factor of PEB (Ulrich, 1984), while recent studies have begun to focus on the cultural and social dimensions of RLP, proving that immersive recreation experiences (Lin and Lee, 2020), spiritual or religious ambience (Lu et al., 2025), and strategically designed environmental education interventions (Yin et al., 2025) can significantly enhance environmental responsibility. RLP, as a holistic construct, integrates these dimensions. It fosters tourists’ environmental responsibility by cognitive-affective integration, which combines emotional resonance with cognitive appreciation of landscapes. Furthermore, the results demonstrated that RLP has a positive impact on place identity (H4), and tourists who perceive recreational landscapes are more likely to establish emotional and cognitive connections with the destination and regard it as part of their self-concept, which is consistent with previous research (Sobhaninia et al., 2023).

The result show that place identity has a significant positive effect on place-specific PEB (H5). Tourists who develop stronger emotional and cognitive connections to a destination are more likely to take environmentally responsible actions on-site, such as properly disposing of waste, following ecological guidelines, and avoiding harm to natural resources. Furthermore, our results confirm that place identification has a significant positive impact on overall PEB (H6). The emotional connections formed during travel transcend the immediate tourist environment and influence tourists’ long-term environmental attitudes and behaviors in their daily lives. Place identity is not limited to situational behavioral effects but also plays a broader role in fostering sustainable habits. Overall, these findings highlight place identify as a key internal mechanism linking environmental stimuli to both place-specific and general PEB, providing strong empirical support for its central role in the extended S-O-R framework.

The mediation analysis further confirmed three indirect pathways: place identity significantly mediates the relationship between RLP and general PEB (H7); place identity mediates the effect of RLP on place-specific PEB (H8); and RLP first enhances place identity, which then increases place-specific PEB, and these immediate protective actions further spill over into general PEB (H9). These pathways link RLP with PEB through place identity and its subsequent behavioral responses, confirms the complex psychological mechanisms underpinning environmental behaviors, affirming its function as an important psychological driver in translating embodied landscape experiences (Biglin, 2020) into tangible conservation actions. This result enhances our understanding of how immersive engagement and deep emotional attachment to place can help inspire environmental self-efficacy (Sh et al., 2022), ultimately encouraging proactive conservation behavior. Therefore, our findings advocate the integration of psychological insights such as place attachment, identity formation, and emotional bonding into environmental policies and destination management practices. Destinations can adopt psychologically-informed biophilic design (Barbiero and Berto, 2021) principles to foster a stronger sense of place identity. Such strategies can improve visitor satisfaction and revisit intention, so to encourage sustained pro-environmental commitment.

Research has also confirmed a significant spillover effect of PEB. Place-specific PEB significantly predicts general PEB (H3). Tourists who engage in environmentally responsible actions during their trip are more likely to adopt similar sustainable practices in their daily lives after leaving the destination. In addition, RLP has a positive and significant indirect effect on general PEB through place-specific PEB (H9). A positive perception of recreational landscapes initially triggers protective behaviors on-site, and these actions serve as a foundation for transferring environmental responsibility to non-tourism settings. This is consistent with previous theory, which suggests that when individuals perform pro-environmental behaviors in one context, they are more likely to take similar actions in other settings (Thøgersen, 1999). This phenomenon can be attributed to psychological connections between behaviors and the consistency of the self-concept (Whitmarsh and O’Neill, 2010). The multisensory involvement of RLP enhances this effect (Spangenberger et al., 2024). This study found that perceptions of recreational landscapes strongly influenced general PEB through the mediating effects of place identity and place-specific PEB. Specifically, after experiencing positive RLP in natural tourist area, tourists not only exhibit stronger environmental protection behaviors in the area but also extend this behavioral tendency to their choices once they return to their daily lives. This spillover effect may be attributed to the activation of visitors’ place identity, which in turn strengthens their environmental self-efficacy and sense of responsibility. The spillover effect highlights the significance of environmental education and scenic area management in improving tourists’ overall environmental behavior, indicating that by developing positive perceptions of recreational sites and local identity, broader environmental actions can be effectively promoted.

### 6.2 Theoretical contributions

Building on key concepts from previous studies, this research makes theoretical advancements in four distinct areas. Firstly, this study is the first attempt to test the effect of RLP as destination-level stimulus on PEB. The empirical results show the importance of external stimuli of destinations in PEB research, hence expanding the scope of PEB studies and providing new insights for environmental psychology and landscape architecture. Secondly, using the S-O-R theoretical framework, the study explored place identity as a mediator in the relationship between RLP and PEB, demonstrating place identity’s function as a bridge between these two variables, and offering new empirical evidence for the application of S-O-R theory in environmental psychology. Thirdly, the study confirms that RLP significantly influences both place-specific PEB and general PEB. It also identifies a path connecting recreation landscape perception, place identity, place-specific PEB, and general PEB, thereby validating the spillover effect of PEB in a new framework. (Thøgersen, 1999; Halpenny, 2010). Finally, this study used Bootstrapping for mediation analysis, contributing methodologically to the existing landscape and tourism literature and further establishing the effectiveness of this technique in analyzing complex models.

### 6.3 Practical implications

The research findings highlight the critical role of RLP in promoting place-specific PEB as well as general PEB, which provides important strategic directions for sustainable destination management, emphasizing the importance of paying close attention to visitor experiences in environmental design and destination management (Vaske and Kobrin, 2001; Lewicka, 2011).

First, park managers can evoke emotional resonance in visitors by enhancing the recreational landscape features, which not only enhances their visit experience but, more importantly, stimulates and strengthens their PEBs. For example, managers can enhance visitors’ awareness and appreciation of natural and cultural landscapes by installing additional interpretive signage, optimizing tour routes, and providing environmental education activities (Chiesura, 2004). At the visitor center of Mt. Langya National Park, videos of tourists engaging in recreational activities in the forest are displayed. This not only attracts visitors to explore nature more deeply but also enhances their perception of the recreational landscape. Secondly, landscape designers can utilize well-designed spaces and facilities to promote the perception process. Mt. Langya National Park is famous for the “Drunken Man Pavilion.” Along the scenic routes, many new rest pavilions have been built, which not only provide spaces for recreational activities but also allow visitors to learn about the local culture. These pavilions promote visitors’ interaction and emotional connection with the place, thereby enhancing their environmental responsibility. Furthermore, visitors’ awareness of the importance of environmental protection can be reinforced by introducing eco-friendly design elements, such as using sustainable materials and green technologies (Đorđević et al., 2024). Finally, the mediating role of place identity between RLP and PEB highlights the importance of enhancing tourists’ place identity. For example, sharing visitors’ positive experiences and stories through social media can enhance other visitors’ emotional attachment to these places (Stedman, 2002; Halpenny, 2010).

### 6.4 Limitations and future study directions

Although this study makes contributions both theoretically and practically, there are still some limitations to address. First, the study involves a primarily cross-sectional approach, which makes it challenging to capture the dynamic changes in the effects of RLP and place identity on PEB. Future research could use a longitudinal or cross-sectional approach to more comprehensively understand and validate the universality and evolving trends of these relationships. Second, the S-O-R theoretical model is scalable and can incorporate additional constructs to extend the theoretical framework. For example, the impacts generated by RLPs may differ based on the types of recreational landscapes perceived by tourists. Previous recreation research has categorized recreational activities into different types, such as appreciation-based, consumption-based, and mechanical types (Dunlap and Hefferman, 1975). Future studies could explore the mechanisms of the impact of different types of RLPs on the promotion of PEB. Third, this study explored the mediating role of place identity, but other variables could also be considered as mediators between RLPs and PEB. Future research could consider other variables as mediators or moderators. For example, past studies have indicated that other positive emotions such as awe (Yaden et al., 2019), flow experiences (Kim and Thapa, 2018), and consumer emotions (Su et al., 2020) can be antecedent variables for visitors’ PEB. Future research could assess these potential variables as mediators or moderators to construct a more complete causal model of behavior. This could help researchers and practitioners further clarify the factors influencing tourists’ PEB at nature-based tourist destinations, ultimately contributing to the sustainable development of these destinations.

## Data Availability

The raw data supporting the conclusions of this article will be made available by the authors, without undue reservation.
